# Bullying Interrupted: Victimized Students in Remote Schooling During the COVID-19 Pandemic

**DOI:** 10.1007/s42380-022-00146-6

**Published:** 2022-10-29

**Authors:** Juuso Repo, Sanna Herkama, Christina Salmivalli

**Affiliations:** INVEST Research Flagship Center/Psychology, University of Turku, Turku, Finland

**Keywords:** Peer victimization, Bullying, Cyberbullying, Remote schooling, COVID-19

## Abstract

We investigated how the transition to remote schooling during the COVID-19 pandemic affected the rates of bullying victimization among students in primary and lower secondary education and analyzed how a specific group of students, namely previously victimized students experienced remote schooling. The 2-month school lockdown offered a unique setting to explore the association between increasing Internet use and cyberbullying and reflect on the overlap between traditional bullying and cyberbullying in a new context. The main sample (*n* = 34 771) consisted of 10–16-year-old Finnish students who responded to an online survey during the remote schooling period in spring 2020. The sample was supplemented with data from two previous surveys conducted in the same schools in 2019 (*n* = 43,216) and in 2017 (*n* = 24,727). The prevalence of bullying victimization decreased substantially in all grade levels during the school lockdown. Physical isolation and surge in students’ Internet use did not seem to lead to an increase in cyberbullying. Before-lockdown victimized students evaluated the time in remote schooling more positively than expected: they reported relatively high school liking and more teacher support than other students. The pre-existing gap in school adjustment between victimized and non-victimized students did not increase, but surprisingly, decreased. Our results highlight the notion that the main arena to fight bullying is within in-person interactions in schools.

## Introduction

Although attention to cyberbullying has been growing in media and public discussion, research shows that most bullying still takes place on school premises (Cosma et al., 2020). There is also evidence that most students who are bullied online are bullied offline as well (Lazuras et al., 2017; Olweus & Limber, 2018; Waasdorp & Bradshaw, 2015), and offline bullying tends to generalize to online contexts over time, rather than vice versa (Salmivalli et al., 2013). According to two recent meta-analyses (Guo, 2016; Kowalski et al., 2014), being a target of traditional bullying was the strongest predictor of cyberbullying victimization. The COVID-19 pandemic created an unexpected setting where students moved to remote schooling while also their face-to-face interactions in free time were heavily restricted. The current study used this unique possibility to study the effects of remote schooling on bullying victimization in a situation where the main context of bullying was locked down. We asked, first, how previously victimized students experienced the lockdown and, second, what happened to the overall rates of bullying during remote schooling.

### What Happened to Bullying During the School Lockdowns?

During the COVID-19 crisis and physical isolation, the public concern about increase in cyberbullying fueled up due to a surge in children’s internet activity (see, e.g., Schmidt et al., 2020; Sultana et al., 2021). Typically, journalists and professionals advocate these assumptions without academic evidence. To give an example, a report from AI-startup company L1GHT (L1GHT, 2020) got a lot of international media coverage in 2020 and was cited widely to show a “70% increase in cyberbullying” due to the COVID-19 crisis (see, e.g., Khan, 2020).

According to Olweus (2012, 2016), the public claims about cyberbullying as a growing problem should not be accepted without warrants: so far bullying research has failed to find consensual longitudinal evidence for increasing overall rates of cyberbullying (see, also Smith, 2018; Wolke et al., 2017). In addition, the prevalence rates are typically incomparable across studies due to different definitions and operationalizations for cyberbullying (Kowalski et al., 2019; Olweus, 2012; Olweus & Limber, 2018; Patchin & Hinduja, 2015).

At the onset of the pandemic, several academic scholars predicted an increase in cyberbullying due to the crisis (Barlett et al., 2021; Han et al., 2021; Karmakar & Das, 2021). For example, Barlett et al. (2021), following the general strain theory, hypothesized that increase in psychological strains (COVID-19 experiences) would increase stress, which in turn would lead to antisocial behavior (e.g., cyberaggression). They found tentative support for the theory among US adult population by collecting data on public online discussions.

Vaillancourt et al. (2021) studied bullying rates before and during the pandemic with a population-based randomized design among Canadian students (*n* = 6578). Their results—contrary to many predictions—indicated that students reported far higher bullying rates before than during the pandemic. Regarding general victimization, the reduction was approximately 50% (from 34.3 to 16.9%). Meanwhile, cybervictimization decreased from 13.8 to 11.5%. Another study by the UNICEF Canadian Companion (2020) also indicated a decrease in bullying, despite an increase in online engagement.

Bacher-Hicks et al. (2022) analyzed data from Google Internet searches to examine changing bullying patterns as COVID-19 disrupted in-person schooling. They found that searches for school bullying and cyberbullying dropped about 30–40% as US schools shifted to remote learning in spring 2020. In addition, they found that bullying searches gradually returned to pre-pandemic levels while schools returned to in-person schooling.

A contrasting finding comes from a study conducted by the European Commissions’ Joint Research Centre (Lobe et al., 2021). Surveying 10–18-year-old children in 11 European countries in summer 2020 (*N* = 6195), the study found that 44% of respondents who had experienced “situations of cyberbullying” some time before, reported experiencing them more during the pandemic than before (22% reported less, 34% the same). The results between countries varied from 51 (Germany) to 24% (Slovenia). Notably, the cyberbullying measure used in the study included also single experiences (e.g., receiving nasty messages), which would not necessarily fall under the traditional criteria for bullying (Olweus, 2016).

### Psychosocial Impact of Remote Schooling on Victimized Students

The impact of the COVID-19 crisis was not proportional among the youth. According to a recent systematic review covering 61 studies on COVID impact on children and adolescents (Panchal et al., 2021), the pandemic has exacerbated the existing social inequities and affected especially previously vulnerable groups (e.g., those with problems in mental health before the pandemic). One possible vulnerable group, which is yet less studied, is students suffering from bullying victimization in school.

Previous studies offer ground to understand why school lockdown possibly created a substantial risk for previously victimized students. First, students who are victimized at school also experience loneliness, anxiety, and depression (Moore et al., 2017; Reijntjes et al., 2010). Second, several studies have shown that bullying victimization is related to weaker school adjustment including self-reported school liking, academic motivation, and academic achievement (Eriksen et al., 2014; Salmivalli et al., 2012). Furthermore, bullying victimization has been found to be associated with difficulties in learning (Berchiatti et al., 2021), and cyberbullying specifically is a risk for reduced school satisfaction (Kowalski et al., 2019). Third, victimized students typically suffer from low-quality family relations (Nocentini et al., 2019), and support from parents became more important during remote schooling (Goman et al., 2021; Panchal et al., 2021). Fourth, positive peer relations and experienced peer support buffered against the negative effects of physical isolation during the COVID pandemic, especially among adolescents (Juvonen et al., 2021; Magson et al., 2021).

During remote schooling, the context of these psychosocial factors was rapidly transformed into a new setting. To our knowledge, no studies have yet examined how the before-lockdown victimized students experienced physical isolation and remote schooling. Online learning will likely remain a central piece of the public education system for the foreseeable future, and thus preventing the achievement gaps from widening amid digital transformation is a key question for research and educational policy around the globe. During this transition, also the peer relationships and bullying processes specifically should be paid attention to.

### Finnish Context

In Finland, basic education is provided in a single structure system (grades 1–9), which corresponds to primary (grades 1–6) and lower secondary education (grades 7–9). It generally starts in the year in which children turn seven. Basic education is compulsory and free of charge, and over 98% of schools are maintained by public municipalities. During the first wave of the COVID-19 pandemic, the Emergency Power Act and national lockdown were enforced by the Finnish government. The school lockdown began on 18 March 2020 and ended on 14 May 2020 lasting 8 weeks in total. During the lockdown, almost all students were schooling from home, and only very few students with special education needs still attended lessons in person. In practice, almost all public and private facilities for children, including sports clubs and other hobbies, were closed, and families were advised to avoid meeting friends or relatives face-to-face.

With a well-developed educational system and academically educated teachers (Ustyn & Eryilmas, 2018), as well as digitally equipped and active children and adolescents (Smahel et al., 2020), Finland was assumably better prepared to continue schooling online compared to many other countries. Delivery of remote education and online classrooms were put in place rapidly (Finnish National Agency for Education, 2020). A great majority of children could access the Internet to keep up with school and social interactions.^1^ According to a during-lockdown student survey (*n* = 48,338), 92% of pupils in basic education were given lessons via video conferencing (Repo et al., 2020). In addition, the pandemic was not the most extreme in Finland, and infections did not reach most Finnish families or schools during the first wave of the pandemic. The rate of confirmed infections was only 2,6% in the Finnish population by October 2021 (Our World in Data, 2021). Thus, Finland can be considered an interesting context to explore students’ adjustment to this rapid pedagogical transformation.

In the broad picture, there has been a consistent decreasing trend in bullying victimization in Finland since 2009, measured biennially among 15–16-years-olds with a large national sample by the School Health Promotion Study (Finnish Institute for Health and Welfare, 2021). The rate of students being bullied at least weekly has dropped from 8.4 (2009) to 5.5% (2019) in 10 years. During these years, over 90% of Finnish basic education schools have implemented the KiVa anti-bullying program, and there have been major reforms in the national school curriculum and student welfare services. Despite strong efforts and evident success, over 5% of students continue to be bullied every week, forced to face their perpetrators in person every school day (Finnish Institute for Health and Welfare, 2021).

### Current Study

The present study concentrated on students in Finnish primary and lower secondary education during the school lockdown and the first wave of the pandemic in spring 2020. The study seized the unique opportunity in time to explore the question of what happens to school bullying when its offline context is locked down, and the only context left to communicate with peers is online. The study aimed to reflect the concerns on ever-increasing rates of cyberbullying and the overlap of traditional and cyberbullying in a new setting. Societal lockdown, physical isolation, and thus the surge in students’ Internet use were at its peak during the first wave of the pandemic (Sultana et al., 2021). Thus, we could assume that almost all—if not all—communication between peers from school took place online during the time of remote schooling.

Further, the study investigated how before-lockdown victimized students experienced remote schooling, focusing on their experiences of school adjustment and perceived support from parents, teachers, and peers. Instead of relying merely on the during-lockdown survey, this study used two other large-scale survey data gathered from the same schools before the pandemic.

## Method

### Participants

The main data of the study came from a large student survey conducted during the school lockdown in spring 2020 (hereafter School lockdown survey, *n* = 34 771), including participants from 406 Finnish public basic education schools (~ 20% of all basic education schools in Finland). Participants were from grade levels 4–9 (10–16-year-olds). All schools implementing the KiVa anti-bullying program (see for more Herkama et al., 2017) in Finland were invited to participate in the survey instead of the annual KiVa survey. Teachers advised students to fill in the anonymous online questionnaire during distant learning classes or as homework. The data were gathered in the first two weeks of May in 2020, 7–8 weeks after the school lockdown had started. The schools reopened nationwide on May 14, and Finnish primary schools have not been in lockdown thereafter.

The School lockdown survey data were supplemented with data from two previous cross-sectional student surveys collected in the same schools (see Table 1). They provide a reference point to the results even though individual respondents could not be matched between the samples. The KiVa survey is conducted annually in all grade levels in schools implementing the KiVa anti-bullying program. The KiVa survey data used in this study were collected in spring 2019 (*n* = 43 216, 335 matching schools).

**Table 1:** Measures for victimization and cybervictimization in different surveys at various time points

Measure	Survey	Time of data collection	Item
During-lockdown victimization	School lockdown survey	May 2020	“Have your peers from school bullied you during the remote schooling?”
Before-lockdown victimization	School lockdown survey	May 2020	“Have your peers from school bullied you during this year, before the remote schooling began?”
General victimization 2019	KiVa Survey	May 2019	“How often have you been bullied at school during the past few months?”
Cybervictimization	KiVa Survey	May 2019	“Have you been bullied through the Internet during the past few months?”
General victimization 2017	SHP Survey	Autumn 2017	“How often have you been bullied in school during the current school year?”

The national School Health Promotion (SHP) Study is a biennial comprehensive student survey conducted in Finnish schools nationwide. It consists of two versions, one for grade levels 4–5 and another for grade levels 8–9. Grades 6–7 do not participate in this survey at all. The subsample with matching schools with the School lockdown survey includes respondents from 249 schools from grades 4–5 (*n* = 13 735) and from 100 schools from grades 8–9 (*n* = 10 992). These samples were collected in Autumn 2017. In addition, previously published national results from the SHP Study were used to assess for a possible bias in the study samples. Table 1 summarizes the study samples and different measures for peer victimization in the surveys.

### Measures in the School Lockdown Survey 2020

Measures for the School lockdown survey were adapted from previous studies, except the measures for parental, teacher, and peer support during the lockdown, which were designed for the present study. The School lockdown survey included no questions on individual demographics except student’s grade level to provide full anonymity and confidentiality for the respondents. This was justifiable given the fact that respondents were informed that school-specific results from the survey would be shared to participating schools immediately after the data collection (Repo et al., 2020).

The School lockdown survey included two single item measures for peer victimization, adapted originally from the Olweus Bully/Victim Questionnaire. First, *during-lockdown peer victimization* was assessed with a global item: “Have your peers from school bullied you during the remote schooling?”. Secondly, *before-lockdown peer victimization* was measured by asking respondents to assess their experiences retrospectively: “Have your peers from school bullied you during this year, before the remote schooling began?”. Response options for both items were “not at all,” “once or twice,” “2 or 3 times a month,” “about once a week,” and “several times a week.”

#### Difficulties in Learning

were measured with a 6-item measure, corresponding to the SHP study. The question “Have you had difficulties with some of the following school-related issues?” included items “following the teaching in online classes,” “doing assignments,” “tasks requiring writing,” “tasks requiring reading,” “tasks requiring calculating,” and “equipment or software related to distance learning” with response options “not at all,” “quite little,” “quite a lot,” and “a lot.” Cronbach’s alpha was 0.88.

#### School Liking

was measured with a question “How do you like schooling at the moment?” with response options “not at all,” “quite little,” “quite a lot,” and “a lot,” corresponding to the SHP study.

#### Anxiety

was measured with the 7-item Generalized Anxiety Disorder Scale, which has been validated among Finnish adolescents (Tiirikainen et al., 2019). The GAD-7 scale includes seven items based on seven core symptoms and inquires the frequency with which respondents suffered from these symptoms within the last 2 weeks. Item responses were rated along a 4-point scale, ranging from 0 (not at all) to 3 (almost every day). Cronbach’s alpha was 0.92.

#### Loneliness

was measured with a single item: “Do you feel lonely?”. For grade, levels 4–6, the response options were “never,” “sometimes,” and “often,” and for grades 7–9, they were “never,” “very rarely,” “sometimes,” “quite often,” and “constantly.” This was because we wanted to keep the response options identical to those in the national SHP Study for the corresponding grades.

#### Perceived Parental Support

was measured by asking “When you think about adult(s) at your home, how much of the following has taken place during the remote schooling?”. The two items were “I have got support and help from them” and “We have had arguments” with response options “less than before,” “same as before,” and “more than before.”

#### Perceived Teacher Support

was measured with two questions. For the question “Do you think there has been something positive in the remote schooling?”, the respondents selected all options that applied to them, from a pre-defined list of options. For the teacher support measure, the option “I have got more support from teachers” was used (1 = yes, 0 = no). The second question was “How did your teacher organize the remote schooling?” with items “By sending us assignments and material,” “By giving out video lessons,” “By talking with me one-to-one,” and “By organizing group discussions in addition to lessons.” (1 = yes, 0 = no).

#### Perceived Peer Support

was measured with the question: “Think about other students in your school and consider how the following applies to you” with items “We have helped each other in schoolwork” and “It has been relieving not to meet peers from school.” The response options ranged from 0 = “not at all” to 4 = “a lot.”

### Measures in the KiVa Survey 2019

#### General Peer Victimization

was measured with a single item adapted from the Olweus Bully/Victim Questionnaire “How often have you been bullied at school during the past few months?” with similar response options to the School lockdown survey.

#### Cybervictimization

was measured with a single item “Have you been bullied through the Internet during the past few months? Cyberbullying can be manifested, for example, as threatening, mocking, spreading rumors and pictures, and intentional exclusion from social groups on different online applications and services (e.g., Instagram, WhatsApp, Snapchat, Twitter, email, discussion forums). The response options were similar to the single-item measure on peer victimization.

### Measures in the School Health Promotion Survey (SHP) 2017

#### General Peer Victimization

was measured with a single item measure from the global HBSC Study: “How often have you been bullied in school during the current school year?” with response options “several times a week,” “about once a week,” “not so often,” and “not at all.”

#### Difficulties in Learning

were measured similarly to the School lockdown survey. The only differences were the words “distance” in the last item and “online” in the first item, which were only included in the School lockdown survey. The Cronbach’s alpha for the measure in the SHP sample was 0.87.

*School liking*, *anxiety*, and *loneliness* were measured identically to the School lockdown survey. The Cronbach’s alpha for anxiety (GAD-7) was 0.92.

### Analytic Plan

The prevalence of peer victimization and cybervictimization were calculated by using the cutoff point “about once a week” being the lower-bound value in all victimization measures. Another typical cutoff option “2–3 times a month” (see Solberg & Olweus, 2003) was not applicable, as it was not used in the SHP study. The chosen cutoff point was also better for differentiating between before- and during-lockdown victimization. The cut-off point was used to divide respondents into groups of victimized and non-victimized students in all samples.

For the analyses comparing results from two different surveys, subsamples were created to include only the respondents from schools represented in both survey samples. In order to test the differences between victimized and non-victimized students on dependent variables, a series of unpaired *t* tests were applied. This was done within each sample. Differences between the samples were not statistically tested. Levene’s test of homogeneity revealed that the variances for the two groups in comparison were not equivalent for any of the dependent variables (*p* < 0.05). Thus, Welch *t* tests were applied to test the differences between the groups. To assess the magnitude of the differences between the groups, Cohen’s *d* effect size was used. In the case of single-item measures, Chi-square tests were used to test whether the group differences were statistically significant (*p* < 0.05). Missing values were treated with listwise deletion.

## Results

Descriptive statistics and bivariate correlations for examined variables are presented in Table 2.

**Table 2:** Descriptive statistics and bivariate correlations for key variables in the School lockdown survey (*n* = 34,771)

Variable	Scale	*n*	*M*	*SD*	(1)	(2)	(3)	(4)	(5)	(6)	(7)	(8)	(9)
During-lockdown victimization (1)	1–5	34,771	1.11	.46	1.00								
Before-lockdown victimization (2)	1–5	34,771	1.29	.71	.50*	1.00							
School liking (3)	1–4	34,771	2.63	.79	−.05*	−.05*	1.00						
Difficulties in learning, grades 7–9 (4)	0–3	16,072	.72	.64	.11*	.14*	−.41*	1.00					
Loneliness, grades 7–9 (5)	1–5	16,072	2.29	1.10	.15*	.26*	−.18*	.25*	1.00				
Anxiety, grades 7–9 (6)	0–3	16,072	.72	.77	.15*	.21*	−.34*	.47*	.49*	1.00			
Got support or help from parents (7)	0–2	34,286	1.29	0.54	−.04*	−.04*	.10*	−.07*	−.10*	−.10*	1.00		
I got more support from teachers (8)	0–1	34,771	.17	.37	.04*	.04*	.13*	−.03*	−.06*	−.06*	.07*	1.00	
We have helped each other in schoolwork (9)	0–4	34,771	1.87	1.18	−.07*	−.11*	.10*	−.08	.00	.00	.04*	−.04*	1.00

**Fig. 1:**
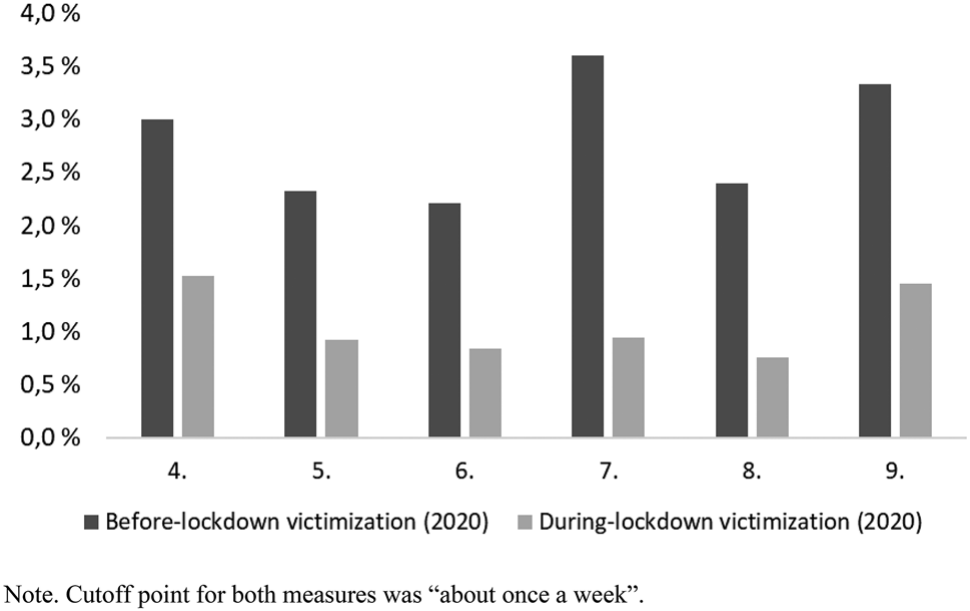
Prevalence of before-lockdown and during-lockdown victimization by grade level

We compared the prevalence of bullying victimization before and during school lockdown, both measured with the School lockdown survey (*n* = 34 771, 406 schools). As can be seen from Fig. 1, the prevalence of bullying victimization was substantially lower in all grade levels during the lockdown. The relative difference in prevalence was 49.3–73.9%, depending on grade level. On average, every third student (out of those previously victimized) continued to be victimized during the lockdown.

However, still, during the remote schooling, 1.1% of students were victimized on a weekly basis and 2.1% at least several times a month. Notably, those who were most frequently bullied before the lockdown were more prone to suffer from continuing victimization during the lockdown. Out of the during-lockdown victimized students, 16.7% were new victims (i.e., had not been victimized at least weekly before the lockdown)—they represented only 0.18% of the whole sample.

**Fig. 2:**
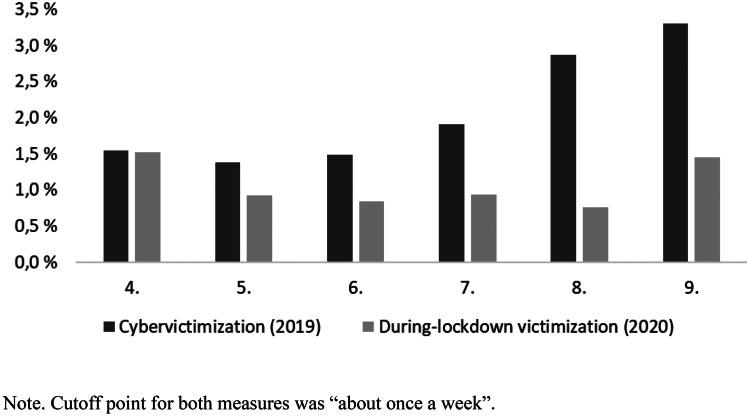
Prevalence of cybervictimization in 2019 and during-lockdown victimization in 2020 by grade level. Visual comparison of two different samples

Further, we compared the prevalence of cybervictimization from KiVa Survey 2019 to the level of during-lockdown victimization in 2020 (see Fig. 2). The comparison does not rely on statistical testing but is merely a visual one, as the samples could not be matched on the individual level. The victimization during the lockdown was assumed to take place mostly online due to the physical isolation. In the 335 schools under study, the average rate of cybervictimization was 2.0% (*n* = 43,216) in 2019 compared to during-lockdown victimization 1.0% (*n* = 28,567). The difference was greatest in higher grade levels—in grade eight (2.9% in 2019 and 0.8% in 2020) and in grade nine (3.3% in 2019 and 1.5% in 2020). As illustrated by Fig. 2, students in upper grade levels (7–9), compared to lower ones (4–6), were more likely to be victimized before the pandemic, but not anymore during the pandemic.

### Adjustment of Before-Lockdown Victimized Students

Further, we examined how the before-lockdown victimized students (*n* = 971, 2.8%) experienced remote schooling by comparing them with non-victimized students (*n* = 33 800, 97.2%) on several outcomes. In the following, by “victimized students,” we refer to the group of students who reported retrospectively that they were victimized before the school lockdown. That is because we were interested in exploring how the change in schooling circumstances affected the adjustment of those previously victimized students.

First, the groups of before-lockdown victimized and non-victimized students were compared on the measures of school liking, difficulties in learning, anxiety, and loneliness. The SHP data from 2017 was used to reflect how similar groups (victimized and non-victimized students) differed during normal education on the same measures. The SHP dataset consisted of two subsamples (students in grades 4–5 and in grades 8–9), and the School lockdown sample was matched to include the corresponding grade levels from the same schools.

According to Table 3, the victimized students had more difficulties in learning, lower school liking, and they felt more anxious and lonelier in comparison to other students in both time points. Thus, cross-sectionally, victimized students were more troubled with school adjustment, expectedly. The only exception was in during-lockdown school liking among 4–5 graders: the gap between victimized and non-victimized that existed before the pandemic was not present during the lockdown (see Table 3).

**Table 3:** Independent group comparisons on four adjustment measures among victimized and non-victimized students during and before remote schooling

		School lockdown survey (2020)									SHP survey (2017)							
		Before-lockdown victimized ͣ			Non-victimized						Victimized			Non-victimized				
	Scale	*n*	*M*	*SD*	*n*	*M*	*SD*	*t*	*d*		*n*	*M*	*SD*	*n*	*M*	*SD*	*t*	*d*
Difficulties in learning, grades 8–9	0–3	197	1.15	.82	7046	.81	.65	5.73	.46		649	1.30	.87	10,030	.87	.63	12.32	.56
Anxiety, grades 8–9	0–3	197	1.26	1.00	7046	.74	.78	7.22	.58		637	1.18	1.02	9997	.50	.64	16.61	.80
School liking, grades 4–5	1–4	240	2.69	.90	8803	2.67	.77	.27	.02		959	2.78	.76	12,619	3.00	.65	−8.59	−.31
School liking, grades 8–9	1–4	197	2.37	.94	7046	2.57	.81	−2.94	−.23		656	2.32	.82	9979	2.64	.67	−9.58	−.42
Loneliness, grades 4–5	1–3	240	2.03	.67	8803	1.45	.56	13.40	.94		953	1.90	.61	12,571	1.31	.50	29.40	1.07
Loneliness, grades 8–9	1–5	197	3.26	1.26	7046	2.32	1.10	10.35	.79		637	3.04	1.41	9952	2.05	.97	17.29	.81

Further, we assessed the difference between samples from 2017 and 2020 to explore whether the pre-existing gap in adjustment between victimized and non-victimized students got wider during the school lockdown. By comparing the results from 2017 and 2020, we can see that both victimized and non-victimized students reported higher levels of loneliness during the lockdown compared to normal conditions in 2017 (see Table 3). The same applied to anxiety, even though the increase in anxiety was greater among the non-victimized (mean difference + 0.24) compared to victimized students (mean difference + 0.08). Surprisingly, both groups—victimized and non-victimized—reported fewer difficulties in learning during the lockdown.

Further, the victimized students in grades 8–9 reported higher school liking during the lockdown than in 2017 (mean difference + 0.05), in contrast to non-victimized students who reported lower school liking during the lockdown (mean difference −0.07). Regarding students in lower grades (4–5), both groups reported weaker school liking, but it decreased less among the victimized students (mean difference −0.16) compared to non-victimized (mean difference −0.32).

As mentioned, cross-sectionally the victimized students continued to have more troubles in adjustment during the lockdown, in comparison to non-victimized students. However, comparing the effect sizes (Cohen’s *d*) from the Welsch *t* tests yields that all the differences between these two groups were smaller during remote schooling (see Table 3). This applied especially to school liking, both among 4–5 graders (before-lockdown *d* =  −0.31, during-lockdown *d* = 0.02), and among 8–9 graders (before-lockdown *d* =  −0.42, during-lockdown *d* =  −0.23). Taken together, the victimized students continued to have weaker school adjustment during the remote schooling, but the gap between the victimized and non-victimized students seemed to decrease.

Further, we looked at the perceived parent, teacher, and peer support by asking the respondents to compare the situation before and during remote schooling, measured in the School lockdown survey. Victimized students reported more decrease in support from parents (χ2(8, *n* = 34 286) = 96.97, *p* < 0.001) and more increase in arguments with parents (χ2(8, *n* = 33 892) = 111.71, *p* < 0.001), compared to other students (see Table 4).

**Table 4:** Parental and teacher support during remote schooling among before-lockdown victimized and non-victimized students

		Victimized		Non-victimized				
Variable	Response options	*n*	%	*n*	%	*χ2*	*df*	*p*
Got support or help from parents						96.97	2	.000
	Less than before	96	10.1%	1287	3.9%			
	Same like before	529	55.8%	20,922	62.8%			
	More than before	523	34.1%	11,129	33.4%			
	Total	948	100%	33,338	100%			
Arguments with parents						111.71	2	.000
	Less than before	243	26.0%	11,482	34.9%			
	Same like before	499	53.3%	18,044	54.8%			
	More than before	194	20.7%	3430	10.4%			
	Total	936	100%	32,956	100%			
How did the teacher organize remote schooling?								
	Sent assignments and materials	971	93.5%	32,786	94.8%	2.86	1	ns
	Held video lessons	971	87.6%	32,785	92.2%	25.67	1	.000
	Talked with me one-to-one	971	40.7%	32,785	36.8%	5.86	1	.015
	Organized group discussions outside lessons	971	38.5%	32,785	41.0%	2.36	1	ns
Was there something positive in remote schooling?								
	I got more support from teachers	971	23.1%	32,785	16.5%	29.20	1	.000
	There was less bullying	971	80.2%	32,785	43.0%	529.71	1	.000
	It was easier to concentrate	971	51.1%	32,785	49.0%	1.48	1	ns

In contrast, regarding teacher support, previously victimized students evaluated the time in remote schooling more positively than other students. They had more one-to-one discussions with their teacher (χ2(1, *n* = 34,771) = 5.86, *p* < 0.015) and were more prone to feel that they got more support than usually from their teachers (χ2(1, *n* = 34,771) = 29.20, *p* < 0.001), compared to other students. To test for possible confounding factors for this association, we ran a logistic regression model controlling for difficulties in learning, school liking, and grade level. The association between before-lockdown victimization and one-to-one teacher support remained significant.

Further, before-lockdown victimized students reported less social support from their peers than other students (Table 5). They received less support in schoolwork from their classmates (*t* =  −8.89, *p* < 0.001) and discussed less with their peers outside lessons (*t* =  −9.02, *p* < 0.001). In addition, not seeing their classmates during the lockdown felt as a relief for half (50.1%) of the before-lockdown victimized students.

**Table 5:** Peer support during remote schooling among before-lockdown victimized and non-victimized students

		Before-lockdown victimized			Non-victimized					
Item	Scale	%	*M*	*SD*	%	*M*	*SD*	*t*	*df*	*p*
We have helped each other in schoolwork	0–4	47.2	1.50	1.31	62.8	1.88	1.17	-8.89	1014.92	.000
I have discussed with my classmates outside lessons	0–4	58.1	1.89	1.37	73.9	2.29	1.22	-9.02	1014.25	.000
It has been a relief not to see my classmates	0–4	50.1	1.74	1.56	18.9	.72	1.09	20.16	997.70	.000

%: proportion of respondents who responded “somewhat,” “a lot” or “very much.” Results from the School lockdown survey (*n* = 34 771)

To assess the representativeness of our main sample and the risk of bias in our study, we compared the prevalence of victimization in all samples (School lockdown survey, SHP, KiVa) including only the matched schools and contrasted the prevalence in each sample to the SHP Study encompassing all schools in Finland in 2017 and 2019 and thus referred as the national SHP (see Table 6). The prevalence of victimization in the study schools in 2017 did not differ substantially from all schools in the same SHP survey from 2017. However, the rates in KiVa survey (2019) and especially the rates in School lockdown survey (2020) seem substantially lower compared to national SHP results from 2017.

**Table 6:** Prevalence of peer victimization in grades 4–5 and in grades 8–9, results from the three study samples and from the national School Health Promotion Study (SHP)

	Grades 4–5		Grades 8–9	
Measure	Current study schools	National SHP schools	Current study schools	National SHP schools
Peer victimization (2017, SHP)	7.0% (13,583)	7.3% (94,957)	6.2% (10,066)	5.8% (72,825)
Peer victimization (2019, KiVa)	5.1% (16,050)	7.2% (98,917)	5.0% (12,509)	5.5% (86,609)
Before-lockdown victimization (2020, school lockdown)	2.7% (9043)	-	2.8% (7243)	-

## Discussion

Our results indicated that the prevalence of school bullying decreased significantly during the two-month school lockdown in Finland in the spring of 2020. On average, every third before-lockdown victimized student continued to be victimized during the lockdown; most often those who were more frequently victimized before the lockdown. Furthermore, there were very few new victims during the 8-week lockdown period. Taken together, and somewhat sadly, the school lockdown seemed to be the most effective universal anti-bullying intervention ever documented. Our results are in line with other recent reports showing a reduction in bullying victimization during the remote schooling (UNICEF, 2020; Vaillancourt et al., 2021; Yang et al., 2021).

Furthermore, our results corroborate with the arguments proposed by Olweus (2012, see also Olweus & Limber, 2018): one should be cautious with headlines on increasing levels of cyberbullying. It seems to be a general and persistent claim that cyberbullying is increasing as children spend more time online: e.g., “Screen time is up – and so is cyberbullying” (Khan, 2020). The school lockdown offered a unique setting to evaluate this claim, and the present study found no support for it. Although students were online more during than before the school lockdown (Schmidt et al., 2020; Sultana et al., 2021), this did not result in higher prevalence of bullying or cyberbullying among peers from school: the prevalence of overall during-pandemic victimization was lower compared to cybervictimization in the before-pandemic setting.

The results point to the direction that cyberbullying is not simply a consequence of new technology but a new form of a long-established problem. Even if exposure to inappropriate messages in general may increase with more time spent online (Lobe et al., 2021), we stress that cyberbullying among peers from school is typically compounded and fueled by an in-person component (Cosma et al., 2020; Olweus, 2012; Waasdorp & Bradshaw, 2015), and thus, it should be seen as a trans-contextual phenomenon, involving both online and offline episodes (Lazuras et al., 2017). Further, it evolves from the same motivational grounds as traditional bullying: the need to gain group status (Pellegrini, 2002; Salmivalli & Peets, 2009) and the need to belong (Roland & Idsøe, 2001; Solomontos-Kountouri & Strohmeier, 2021). Hence, bullying prevention initiatives should primarily focus on bullying holistically, no matter what the medium or context (Chudal et al., 2021; Cosma et al., 2020; Gradinger et al., 2015). Placing strong focus on cyberbullying and maintaining the perception that “all bullying takes place online” might not be the optimal way to reduce bullying since this line of thinking might prevent various stakeholders from intervening in the common underlying mechanisms and the most common forms of bullying (Herkama & Salmivalli, 2018; Olweus, 2012). Claims about cyberbullying epidemic may also lead cyberbullying to be considered normative behavior and lead to feelings of helplessness among school staff and students (Sabella et al., 2013). Previous studies have shown that general anti-bullying programs can be effective in reducing cyberbullying (Williford et al., 2013), even without a specific element focusing on cyberbullying (Gradinger et al., 2015).

The results on bullying and remote schooling highlight the Janus-faced nature of the school system: whereas for many, school represents a supportive structure away from despair, and for others, it is the source of that despair. The substantial reductions of peer victimization from the pre‐pandemic to during pandemic indicate that bullying is a dynamic social problem that evolves in the school community. Theoretically, this can be linked with recent writings by several Nordic scholars (Horton, 2018; Repo & Repo, 2016; Restad, 2020; Søndergaard & Rabøl Hansen, 2018; Thornberg et al., 2018), emphasizing a pedagogical perspective to school bullying, and thus the importance of community-oriented teaching practices, pedagogy, and curriculum in bullying prevention. Bullying researchers have increasingly argued for understanding school as a community that may constrain or reinforce bullying and thus called for integrated approaches to teaching and bullying prevention (for a review see Restad, 2021). Remote schooling was a major transformation for pedagogy, students’ relations, autonomy, and social stressors, which may offer important insights for future studies and policy in this respect.

Our study was among the first to explore how students suffering from bullying victimization experienced remote schooling during the COVID-19 pandemic. We had several reasons to assume that before-lockdown victimized students were at special risk for negative impact of the crisis. However, comparing the results from before and during the pandemic, this seems not to be the case. Despite the fact that victimized students, on average, continued to have worse school adjustment than others, the gap between the victimized and non-victimized students became smaller during the school lockdown. This applied especially among adolescents (14–16-year-olds), among whom the during-lockdown decrease in victimization was more notable, compared to younger students. Before-lockdown victimized adolescents seemed to experience higher school liking and less difficulties in learning during than before the school lockdown. Furthermore, victimized students reported receiving more teacher support compared to other students during the lockdown. Remote schooling offered relief from peer victimization for a great number of students, and it may have offered new realms for teacher-student communication. Blended learning—combining in‐person instruction with online learning—has potential to afford more options to students who do not thrive in traditional classrooms. Indeed, previous studies indicate how decreases in victimization are related to better academic achievement over time (Eriksen et al., 2014; Salmivalli et al., 2012). Examining new educational practices, which may mitigate bullying, is of key importance.

Our results also highlight the importance of pre-pandemic data to assess the COVID impact on youth. The majority of studies about the COVID impact are based on single-time cross-sectional surveys, often echoing the salient concerns of public discussion. Without the pre-pandemic data from 2017 and 2019, our data from 2020 would have suggested that victimized students were worse off during the lockdown than other students and the notion that the pre-existing gap in school adjustment to other students actually decreased due to the lockdown would have not been revealed. To understand the mixed effects of the COVID pandemic on children and youth, more longitudinal and qualitative studies are required.

### Strengths and Limitations

The present study has a number of strengths. It relies on a large student sample collected during the exceptional time of global pandemic and school lockdown, while also utilizing previous large data sets from the same schools with similar measures. This enabled the comparisons between pre-pandemic and during-pandemic conditions. Studies on the COVID impact on children and adolescents’ adjustment rely typically on single-time cross-sectional data. Furthermore, there are very few studies on bullying during remote schooling.

Despite these strengths in the study design, there are several limitations to consider. One clear limitation is that we could not match the individual respondents from the pre-pandemic surveys with the during-pandemic survey. Given the study design was merely pseudo-longitudinal, causal conclusions cannot be drawn. Although all three samples are large and gathered from the same schools at corresponding grade levels, the during-lockdown sample may not be as representative as the other samples. School staff may have had troubles in reaching or motivating all students to participate. Unfortunately, we did not have respondent’s gender or other demographics available to analyze possible bias in the during-lockdown sample. In addition, we do not know the exact response rate of the surveys, as they were gathered through schools, and we do not know which classes did not take part in the survey. Thus, the sample differences in bullying prevalence may reflect an actual change in schools, or it may be due to the differences in the samples.

Further, we relied on students’ retrospective assessments on whether they were victimized before the lockdown. However, given the salience of bullying, we suspect that victimization is not likely to be easily forgotten. In addition, the victimization measures were not completely identical in all surveys. For example, the timeframe for victimization was not equivalent in the pre‐pandemic and during-pandemic conditions, which could have inflated pre‐pandemic results because students had a longer period to consider. On the other hand, we did not measure during-lockdown cybervictimization explicitly, but assumed that during-lockdown victimization took place (at least mostly) online, due to extreme societal lockdown and restrictions for in-person interaction in Spring 2020. While a few students may still have met their peers in-person, the actual rates of during-lockdown cybervictimization may have been even lower than our results suggested. Furthermore, all victimization measures consisted of single items, which was the case in several other measures as well. Despite its limitations, the one global item assessing bullying prevalence has proven functional in terms of construct validity and psychometric properties (Solberg & Olweus, 2003).

Our study is limited to the first wave of the COVID-19 pandemic and 2-month school lockdown in Finland. Even though the societal lockdown has not been as total since, at least not in Finland, the effects of physical isolation and remote schooling may have developed in time. In countries where school lockdowns have been longer, rates of cybervictimization may have been higher. Importantly, years to come will show how remote schooling and the pandemic in general has influenced the rates of bullying. It remains to be explored whether the rates will return to the same level as before pandemic or not and whether factors such as length of remote schooling were connected to these changes. Thus, further research is needed to understand the overall impact of the pandemic and remote schooling to bullying, victimized students and other vulnerable groups in different cultural context and over time.

## Data Availability

Data from the School lockdown survey is available from the first author upon request.

## Footnotes

1 According to a recent EU KIDS ONLINE 2020 study, 97% of Finnish 9–16-year-olds have access to smartphone and 85% are online daily (Smahel et al., 2020).

## References

[CR1] Bacher-Hicks A, Goodman J, Green JG, Holt MK (2022). The COVID-19 pandemic disrupted both school bullying and cyberbullying. American Economic Review: Insights.

[CR2] Barlett CP, Rinker A, Roth B (2021). Cyberbullying perpetration in the COVID-19 era: An application of general strain theory. The Journal of Social Psychology.

[CR3] Berchiatti, M., Ferrer, A., Galiana, L., Badenes-Ribera, L., & Longobardi, C. (2021, July). Bullying in students with special education needs and learning difficulties: The role of the student–teacher relationship quality and students’ social status in the peer group. In *Child & Youth Care Forum* (pp. 1–23). Springer US. 10.1007/s10566-021-09640-2

[CR4] Chudal, R., Tiiri, E., Brunstein Klomek, A., Ong, S. H., Fossum, S., Kaneko, H., & Sourander, A. (2021). Victimization by traditional bullying and cyberbullying and the combination of these among adolescents in 13 European and Asian countries. *European child & adolescent psychiatry*, 1-14. 10.1007/s00787-021-01779-6 10.1007/s00787-021-01779-6 PMC9402766 33884501

[CR5] Cosma A, Walsh SD, Chester KL, Callaghan M, Molcho M, Craig W, Pickett W (2020). Bullying victimization: Time trends and the overlap between traditional and cyberbullying across countries in Europe and North America. International Journal of Public Health.

[CR6] Eriksen TLM, Nielsen HS, Simonsen M (2014). Bullying in elementary school. Journal of Human Resources.

[CR7] Finnish Institute for Health and Welfare. (2021). *Kouluterveyskyselyn tulokset* [Results from the School Health Promotion Study]. Finnish Institute for Health and Welfare. Retrieved October 28, 2022, from http://www.thl.fi/kouluterveyskysely

[CR8] Finnish National Agency for Education. (2020). *Distance education in Finland during the COVID-19 crisis. Initial observations.* Finnish National Agency for Education. Retrieved October 28, 2022, from https://www.oph.fi/sites/default/files/documents/distance-education-in-finland-during-covid19_initial-observations.pdf

[CR9] Goman, J., Huusko, M., Isoaho, K., Lehikko, A., Metsämuuronen, J., Rumpu, N., Seppälä, H., Venäläinen, S., & Åkerlund, C. (2021). *Poikkeuksellisten opetusjärjestelyjen vaikutukset tasa-arvon ja yhdenvertaisuuden toteutumiseen eri koulutusasteilla. – Arviointihankkeen osa III: Kansallisen arvioinnin yhteenveto ja suositukset.* [Impacts of the exceptional teaching arrangements on the realisation of equality and equity at different levels of education. – Part III of the evaluation project: Summary and recommendations of the national evaluation]. Finnish Education Evaluation Centre. Retrieved October 28, 2022, from https://karvi.fi/wp-content/uploads/2021/04/KARVI_0821.pdf

[CR10] Gradinger P, Yanagida T, Strohmeier D, Spiel C (2015). Prevention of cyberbullying and cyber victimization: Evaluation of the ViSC social competence program. Journal of School Violence.

[CR11] Guo S (2016). A meta-analysis of the predictors of cyberbullying perpetration and victimization. Psychology in the Schools.

[CR12] Han Z, Wang Z, Li Y (2021). Cyberbullying involvement, resilient coping, and loneliness of adolescents during COVID-19 in rural China. Frontiers in Psychology.

[CR13] Herkama, S., Saarento, S., & Salmivalli, C. (2017). The KiVa antibullying program: Lessons learned and future directions. In P. Sturmey (Ed.) *The Wiley handbook of aggression and violence*. Volume III: Societal interventions. West Sussex, UK: Wiley. 10.1002/9781119057574.whbva124

[CR14] Herkama, S. & Salmivalli, C. (2018). KiVa antibullying program. In M. Campbell & S. Bauman (Eds.) *Reducing cyberbullying in schools. International evidence-based practices (pp. 125–134)*. London: Elsevier. 10.1016/C2016-0-01087-2

[CR15] Horton, P. (2018). Towards a critical educational perspective on school bullying. *Nordic Studies in Education*, *38*(4), 302–318. 10.18261/issn.1891-2018-04-02

[CR16] Juvonen J, Schacter HL, Lessard LM (2021). Connecting electronically with friends to cope with isolation during COVID-19 pandemic. Journal of Social and Personal Relationships.

[CR17] Karmakar, S., & Das, S. (2021). Understanding the rise of Twitter-based cyberbullying due to COVID-19 through comprehensive statistical evaluation. In *Proceedings of the 54th Hawaii International Conference on System Sciences*. Retrieved October 28, 2022, from http://hdl.handle.net/10125/70923

[CR18] Khan, G. (2020, Oct 2). *Screen time is up—and so is cyberbullying.* National Geographic*. *Retrieved October 28, 2022, from https://www.nationalgeographic.co.uk/science-and-technology/2020/10/screen-time-use-is-up-and-so-is-cyberbullying

[CR19] Kowalski RM, Giumetti GW, Schroeder AN, Lattanner MR (2014). Bullying in the digital age: A critical review and meta-analysis of cyberbullying research among youth. Psychological Bulletin.

[CR20] Kowalski RM, Limber SP, McCord A (2019). A developmental approach to cyberbullying: Prevalence and protective factors. Aggression and Violent Behavior.

[CR21] Lazuras L, Barkoukis V, Tsorbatzoudis H (2017). Face-to-face bullying and cyberbullying in adolescents: Trans-contextual effects and role overlap. Technology in Society.

[CR22] L1GHT. (2020). *Rising levels of hate speech & online toxicity during this time of crisis*. L1ght. Retrieved October 28, 2022, from https://l1ght.com/Toxicity_during_coronavirus_Report-L1ght.pdf

[CR23] Lobe B, Velicu A, Staksrud E, Chaudron S, Di Gioia R (2021). How children (10–18) experienced online risks during the COVID-19 lockdown - Spring 2020. Publications Office of the European Union.

[CR24] Magson NR, Freeman JY, Rapee RM, Richardson CE, Oar EL, Fardouly J (2021). Risk and protective factors for prospective changes in adolescent mental health during the COVID-19 pandemic. Journal of Youth and Adolescence.

[CR25] Moore, S. E., Norman, R. E., Suetani, S., Thomas, H. J., Sly, P. D., & Scott, J. G. (2017). Consequences of bullying victimization in childhood and adolescence: A systematic review and meta-analysis. *World journal of psychiatry*, *7*(1), 60. https://doi.org/10.5498%2Fwjp.v7.i1.60 10.5498/wjp.v7.i1.60 PMC5371173 28401049

[CR26] Nocentini A, Fiorentini G, Di Paola L, Menesini E (2019). Parents, family characteristics and bullying behavior: A systematic review. Aggression and Violent Behavior.

[CR27] Olweus D (2012). Cyberbullying: An overrated phenomenon?. European Journal of Developmental Psychology.

[CR28] Olweus, D. (2016). Cyberbullying: A critical overview. In Bushman, B. (Ed.) *Aggression and Violence: A Social Psychological Perspective* (pp. 225–240). Routledge. 10.4324/9781315524696

[CR29] Olweus D, Limber SP (2018). Some problems with cyberbullying research. Current Opinion in Psychology.

[CR30] Our World in Data. (2021). *Finland: Coronavirus pandemic country profile. *Retrieved October 28, 2022, from https://ourworldindata.org/coronavirus/country/finland

[CR31] Panchal, U., Salazar de Pablo, G., Franco, M., Moreno, C., Parellada, M., Arango, C., & Fusar-Poli, P. (2021). The impact of COVID-19 lockdown on child and adolescent mental health: systematic review. *European child & adolescent psychiatry*, 1-27. 10.1007/s00787-021-01856-w 10.1007/s00787-021-01856-w PMC8371430 34406494

[CR32] Patchin JW, Hinduja S (2015). Measuring cyberbullying: Implications for research. Aggression and Violent Behavior.

[CR33] Pellegrini AD (2002). Bullying, victimization, and sexual harassment during the transition to middle school. Educational Psychologist.

[CR34] Reijntjes A, Kamphuis JH, Prinzie P, Telch MJ (2010). Peer victimization and internalizing problems in children: A meta-analysis of longitudinal studies. Child Abuse & Neglect.

[CR35] Repo, J., Poskiparta, E., Herkama, S., & Salmivalli, C. (2020). *Koululaisten koronakevät: kyselyn tulokset. Interaktiivinen tulostyökalu* [Schoolchildren’s coronaspring: survey results. Interactive results tools]. University of Turku. Retrieved October 28, 2022, from https://bit.ly/koronakevat

[CR36] Repo L, Repo J, Saracho O (2016). Integrating bullying prevention in early childhood education pedagogy. Contemporary perspectives on research on bullying and victimization in early childhood education.

[CR37] Restad, F. (2020). Is there a hole in the whole-school approach? A critical review of curriculum understanding in bullying research. *Nordic Studies in Education*, *40*(4), 362–386. 10.23865/nse.v40.2610

[CR38] Restad, F. (2021). *Curriculum making for social learning. Exploring policy and practice in norwegian lower secondary education.* [Doctoral dissertation in child and youth participation and competence development 2021. Department of Education, Inland Norway university of Applied Sciences]. Retrieved October 28, 2022, from https://hdl.handle.net/11250/2757476

[CR39] Roland E, Idsøe T (2001). Aggression and bullying. Aggressive Behavior: Official Journal of the International Society for Research on Aggression.

[CR40] Sabella RA, Patchin JW, Hinduja S (2013). Cyberbullying myths and realities. Computers in Human Behavior.

[CR41] Salmivalli C, Sainio M, Hodges EV (2013). Electronic victimization: Correlates, antecedents, and consequences among elementary and middle school students. Journal of Clinical Child & Adolescent Psychology.

[CR42] Salmivalli C, Garandeau CF, Veenstra R, Ryan AM, Ladd GW (2012). KiVa anti-bullying program: Implications for school adjustment. Peer relationships and adjustment at school.

[CR43] Salmivalli C, Peets K, Rubin KH, Bukowski WM, Laursen B (2009). Bullies, victims, and bully-victim relationships in middle childhood and early adolescence. Handbook of peer interactions, relationships, and groups.

[CR44] Schmidt SC, Anedda B, Burchartz A, Eichsteller A, Kolb S, Nigg C, Woll A (2020). Physical activity and screen time of children and adolescents before and during the COVID-19 lockdown in Germany: A natural experiment. Scientific Reports.

[CR45] Smahel, D., Machackova, H., Mascheroni, G., Dedkova, L., Staksrud, E., Ólafsson, K., Livingstone, S., & Hasebrink, U. (2020). *EU Kids Online 2020: Survey results from 19 countries*. 10.21953/lse.47fdeqj01ofo

[CR46] Smith PK (2018). The psychology of school bullying. Routledge.

[CR47] Solberg ME, Olweus D (2003). Prevalence estimation of school bullying with the Olweus Bully/Victim Questionnaire. Aggressive Behavior: Official Journal of the International Society for Research on Aggression.

[CR48] Solomontos-Kountouri O, Strohmeier D (2021). The need to belong as motive for (cyber) bullying and aggressive behavior among immigrant adolescents in Cyprus. New Directions for Child and Adolescent Development.

[CR49] Søndergaard, D. M., & Rabøl Hansen, H. (2018). Bullying, social exclusion anxiety and longing for belonging. *Nordic Studies in Education*, 38(4), 319–336. 10.18261/issn.1891-2018-04-03

[CR50] Sultana, A., Tasnim, S., Hossain, M. M., Bhattacharya, S., & Purohit, N. (2021). Digital screen time during the COVID-19 pandemic: A public health concern. *F1000Research*, *10*(81), 81. 10.12688/f1000research.50880.1

[CR51] Thornberg, R., Baraldsnes, D., & Saeverot, H. (2018). Editorial: In search of a pedagogical perspective on school bullying. *Nordic Studies in Education*, 38(04), 289–301. 10.18261/issn.1891-2018-04-01

[CR52] Tiirikainen K, Haravuori H, Ranta K, Kaltiala-Heino R, Marttunen M (2019). Psychometric properties of the 7-item generalized anxiety disorder scale (GAD-7) in a large representative sample of Finnish adolescents. Psychiatry Research.

[CR53] UNICEF Canadian Companion. (2020). *Worlds apart: Canadian companion to UNICEF report card 16*. Retrieved October 28, 2022, from https://oneyouth.unicef.ca/sites/default/files/2020-09/UNICEF%20RC16%20Canadian%20Companion%20EN_Web.pdf

[CR54] Ustyn, U., & Eryilmas, A. (2018). Analysis on Finnish education system to question the reasons behind Finnish success in PISA. *Studies in Educational Research and Development**2*(2), 93–114. Retrieved October 28, 2022, from http://files.eric.ed.gov/fulltext/ED591431.pdf

[CR55] Vaillancourt T, Brittain H, Krygsman A, Farrell AH, Landon S, Pepler D (2021). School bullying before and during COVID-19: Results from a population-based randomized design. Aggressive Behavior.

[CR56] Waasdorp, T. E., & Bradshaw, C. P. (2015). The overlap between cyberbullying and traditional bullying. *Journal of Adolescent Health*, *56*, 483­–488. 10.1016/j.jadohealth.2014.12.002 10.1016/j.jadohealth.2014.12.002 25631040

[CR57] Williford A, Elledge LC, Boulton AJ, DePaolis KJ, Little TD, Salmivalli C (2013). Effects of the KiVa antibullying program on cyberbullying and cybervictimization frequency among Finnish youth. Journal of Clinical Child & Adolescent Psychology.

[CR58] Wolke D, Lee K, Guy A (2017). Cyberbullying: A storm in a teacup?. European Child & Adolescent Psychiatry.

[CR59] Yang, X., Harrison, P., Huang, J., Liu, Y., & Zahn, R. (2021). The impact of COVID-19-related lockdown on adolescent mental health in China: A prospective study. 10.2139/ssrn.3792956

